# Analytical performance of 3 *m* and 3 *m +*1 armchair graphene nanoribbons under uniaxial strain

**DOI:** 10.1186/1556-276X-9-598

**Published:** 2014-11-04

**Authors:** Eng Siew Kang, Razali Ismail

**Affiliations:** 1 Department of Electronic and Computer Engineering, Southern University College, Skudai 81310, Johor Darul Takzim, Malaysia; 2 Department of Electronic Engineering, Faculty of Electrical Engineering, Universiti Teknologi Malaysia, Skudai 81310, Johor Darul Takzim, Malaysia

**Keywords:** Graphene nanoribbons, Uniaxial strain, Bandgap, Carrier density, Drain current

## Abstract

The electronic band structure and carrier density of strained armchair graphene nanoribbons (AGNRs) with widths of *n* =3 *m* and *n* =3 *m* +1 were examined using tight-binding approximation. The current-voltage (*I*-*V*) model of uniaxial strained *n* =3 *m* AGNRs incorporating quantum confinement effects is also presented in this paper. The derivation originates from energy dispersion throughout the entire Brillouin zone of uniaxial strained AGNRs based on a tight-binding approximation. Our results reveal the modification of the energy bandgap, carrier density, and drain current upon strain. Unlike the two-dimensional graphene, whose bandgap remains near to zero even when a large strain is applied, the bandgap and carrier density of AGNRs are shown to be sensitive to the magnitude of uniaxial strain. Discrepancies between the classical calculation and quantum calculation were also measured. It has been found that as much as 19% of the drive current loss is due to the quantum confinement. These analytical models which agree well with the experimental and numerical results provide physical insights into the characterizations of uniaxial strained AGNRs.

## Background

Graphene, as a two-dimensional single layer of carbon in hexagonal symmetry, has attracted considerable attention since being experimentally discovered in 2004. It possesses various fascinating electrical and physical properties, such as extremely high mobility of the charge carrier, high switching speed with ballistic transport behaviors, and anomalous quantum Hall effects [1,2]. These excellent electronic properties make graphene a promising alternative as a building block in potential nanoelectronic devices [3]. To further develop the graphene’s application in field-effect transistors (FETs), various studies have attempted to modulate the electronic structure using mechanical deformation [2,4]. This offers the tempting prospect of controlling the electronic properties of graphene structure by the introduction of strain. The influence of strain on Raman spectroscopy and energy gap of graphene has been predicted theoretically and realized experimentally [5-7]. However, two-dimensional graphene shows zero bandgap electronic properties. Even if a strain as large as 20% is applied, the bandgap remains close to zero. Interestingly, graphene patterned into nanoribbons, referred as graphene nanoribbons (GNRs), has been demonstrated to possess a bandgap opening made possible by tuning the ribbon width [5,8,9]. Finite-width strips GNRs (<10 nm) with quasi 1D structure are expected to present similar electronic properties to graphene and carbon nanotubes. However, the spectrum of GNRs depends on the nature of their edge shapes, namely, zigzag-edge and armchair-edge GNRs (ZGNRs and AGNRs). Nevertheless, ZGNRs have been found to be metallic for all widths, while AGNRs are either metallic or semiconducting, depending on their widths [10,11].

Strain may have a vital influence on further tailoring the electronic properties of a material. Strain in silicon, germanium, and silicon germanium have been successfully implemented by the conventional semiconductor industry, with significant improvements in carrier mobility [12-14]. Understanding the influence of strain on GNRs is of great importance. A substantial part in the fabrication process of GNR device involves deposition of carbon nanostructures on the substrate, which introduces strain at the interface. As GNR area is one-atom thick film, interface strain-induced variations in the electronic and vibrational structures are expected to play a greater role compared to free-standing GNRs. A further motivation to examine the incorporation of strain is the prospect of bandgap opening [15]. Theoretically, the potential of uniaxial strain on the energy gap of GNRs has been widely adopted based on *ab initio* approaches and tight-binding approximation [15-18]. It has been shown that ZGNRs and AGNRs possess distinct energy gap properties under strain. Despite the fact that there have been many studies on the strain effect in graphene and GNRs, most of the previous works focused on the electronic band structure particularly the energy bandgap, while the effect of strain on the carrier density has seldom been studied. Analytical carrier density expressions will find widespread use in determining equilibrium or quasi equilibrium electronics and transport properties in a semiconductor. The carrier density can be determined without having to perform extensive time-consuming numerical simulations. It can also be utilized in the development of fast compact models for circuit simulation. Although the strain effects on the energy bandgap of GNRs has been explored, a comparative study between different families of AGNRs *n* =3 *m* and *n* =3 *m* +1 is still lacking. Therefore, in this paper we theoretically explore the influence of uniaxial strain on the band structures and carrier density of AGNRs for both the *n =*3 *m* and *n =*3 *m +*1 families using tight-binding calculations and formulate a universal explanation for the effect of strain. In addition, we also investigated the effect of quantum confinement on the drain-current performance of *n =*3 *m* AGNRs and compared the analytical results against experimental data.

## Methods

### Theoretical model for electronic properties

In order to investigate the uniaxial strain effect on AGNRs, we adopted the energy dispersion established by Mei et al. [19], which is based on tight-binding approximation. The band energy throughout the entire Brillouin zone of AGNRs is expressed as follows:

(1) $$E\left(k\right)=\pm \sqrt{\left[{t_{1}}^{2}+4{t_{2}}^{2}\cos^{2}\left(\frac{p\pi }{n+1}\right)+4t_{1}t_{2}\cos\left(\frac{p\pi }{n+1}\right)\cos\left(\frac{3}{2}k_{x}a_{\mathrm{cc}}\right)\right]}$$

where *t*_1_ = *t*_0_/(1 + *ϵ*)^2^ and *t*_2_ = *t*_0_/(1 + *ϵ*/4) with *t*_0_ = −2.7 eV are the nearest hopping integral without strain, *a*_*cc*_ =1.42 Å is the carbon-carbon bond length, *k*_*x*_ is the wave vector in the *x-*direction, *n* is the number of dimmer lines across the ribbon widths, and *p* is the band index running from 1 to *n*. The positive and negative prefixes are the band structures for the π* (conduction) and *π* (valence) bands, respectively [20]. Here, *ϵ* denotes as the magnitude of uniaxial strain. The width of AGNRs, *w*, is proportional to *n* given by the following expression:

(2) $$w=\left(n-1\right)\frac{\sqrt{3}}{2}a_{\mathrm{cc}}$$

GNRs' valence and conduction bands are degenerate at six points located on the corners of the Brillouin zone, also called as K and K′ valleys. The electronic properties of GNRs are invariant by interchanging the K and K′ states which mean that the two valleys are related by time-reversal symmetry. The energy separation between the conduction band top and valence band top occurs at Γ points (*k*_*x*_ =0); hence, the energy bandgap results in the following:

(3) $$E_{g}=2\left[t_{1}+2t_{2}\cos\left(\frac{p\pi }{n+1}\right)\right]$$

Using a Taylor expansion to the first order, the energy dispersion relation in Equation 1 can be further approximated as follows:

(4) $$E\left(k\right)=\pm \sqrt{\left[{\left(\frac{E_{g}}{2}\right)}^{2}-\frac{9}{2}t_{1}t_{2}\cos\left(\frac{p\pi }{n+1}\right)a_{\mathrm{cc}}{k_{x}}^{2}\right]}$$

The energy dispersion in Equation 4 shows a nonparabolic relation with the wave vector. For low-lying energy states in which the majority of the electrons are likely to reside, the band structure is approximated to parabolic characteristics using the square root approximation model $\sqrt{1+\alpha^{2}}\approx 1+\alpha^{2}/2,\;\alpha \;\approx \;1$ in a macrochannel and α <1 for a nanochannel. Therefore, the conduction band energy in the low-energy limit is reduced to as follows:

(5) $$E\left(k\right)=\frac{E_{g}}{2}-\frac{9}{2E_{g}}t_{1}t_{2}\cos\left(\frac{p\pi }{n+1}\right){a_{\mathrm{cc}}}^{2}{k_{x}}^{2}$$

### Theoretical model for carrier density

In this section, we further derive the carrier density of AGRNs directly from the density of states (DOS) and Fermi-Dirac distribution in the energy space. In the parabolic part of the energy structure, the DOS reveals the number of available states to be occupied [21]. The DOS of AGNRs can be expressed as follows:

(6) $$D\left(E\right)=\frac{\Delta N}{\Delta E.L}=\frac{1}{4\pi }\sqrt{\frac{E_{g}}{-\frac{9}{2}t_{1}t_{2}\cos\left(\frac{p\pi }{n+1}\right){a_{\mathrm{cc}}}^{2}}}\frac{1}{\sqrt{E-\left(\frac{E_{g}}{2}\right)}}$$

where *N* is the quantum number and *L* is the ribbon length. The DOS of AGNRs under uniaxial strain reveals that the energy states in both low and high regions are affected by the strain.

The carrier density is the fundamental parameter to describe the electrostatics and transport properties of electrons and holes in a semiconductor. The electron carrier density (*n*) is formally given as follows:

(7) $$n=\int_{E_{c}}^{E_{\mathrm{top}}}D\left(E\right)f\left(E\right)dE$$

with

(8) $$f\left(E\right)=\frac{1}{1+e^{E-E_{F}/k_{B}T}}$$

Here, *f*(*E*) is the Fermi-Dirac distribution which describes the degeneracy of the electron concentration, *E*_*F*_ is the Fermi energy, *k*_*B*_ is the Boltzmann constant, and *T* is the ambient temperature in Kelvin. *E*_*top*_ and *E*_*c*_ are the top and bottom of the conduction band, respectively. By making some substitutions for simplicity *x* = (*E* − *E*_*g*_/2)/*k*_*B*_*T* and *η*_*F*_ = (*E*_*F*_ − *E*_*g*_/2)/*k*_*B*_*T*, the carrier density integral is simplified as follows:

(9) $$n=\frac{1}{4\pi }\sqrt{\frac{E_{g}}{-\frac{9}{2}t_{1}t_{2}\cos\left(\frac{p\pi }{n+1}\right){a_{\mathrm{cc}}}^{2}}}k_{B}T\int_{E_{c}}^{E_{\mathrm{top}}}\frac{x^{-1/2}}{e^{x-\eta_{F}}}dx$$

The Fermi-Dirac integral of order *i −* is defined as follows:

(10) $${ℑ}_{i}\left(\eta_{F}\right)=\frac{1}{\Gamma \left(i+1\right)}\int_{0}^{\infty }\frac{x^{i}}{e^{x-\eta_{F}}+1}dx$$

where Γ (*i* +1) is a gamma function. The Fermi integral with Maxwellian approximation is always an exponential for all values of *i* and is given by

(11) $${ℑ}_{i}\left(\eta_{F}\right)\approx e^{\eta }\;\left(\mathrm{nondegenerate}\right)$$

In the strongly degenerate regime, the Fermi integral transforms to

(12) $${ℑ}_{i}\left(\eta_{F}\right)=\frac{1}{\Gamma \left(i+1\right)}\frac{\eta^{i+1}}{i+1}=\frac{\eta^{i+1}}{\Gamma \left(i+2\right)}\;\left(\mathrm{degenerate}\right)$$

Based on the Fermi-Dirac integral approximation described above, the electron carrier density can be obtained as follows:

(13) $$n=\frac{\sqrt{E_{g}k_{B}T}}{4\sqrt{-\frac{9\pi }{2}t_{1}t_{2}\cos\left(\frac{p\pi }{n+1}\right){a_{\mathrm{cc}}}^{2}}}{ℑ}_{-\frac{1}{2}}\left(\eta_{F}\right)$$

The Fermi-Dirac integral of half-order with a closed-form solution in the degenerate regime in which the Fermi level is located 3 *k*_*B*_*T* within the conduction or valence band edges, is given as follows:

(14) $${ℑ}_{-\frac{1}{2}}\left(\eta_{F}\right)=\frac{1}{\sqrt{\pi }}\int_{0}^{\infty }\frac{E^{-1/2}}{1+e^{E-\eta_{F}}}dE=\frac{2{\eta_{F}}^{\frac{1}{2}}}{\sqrt{\pi }}$$

**Figure 1:**
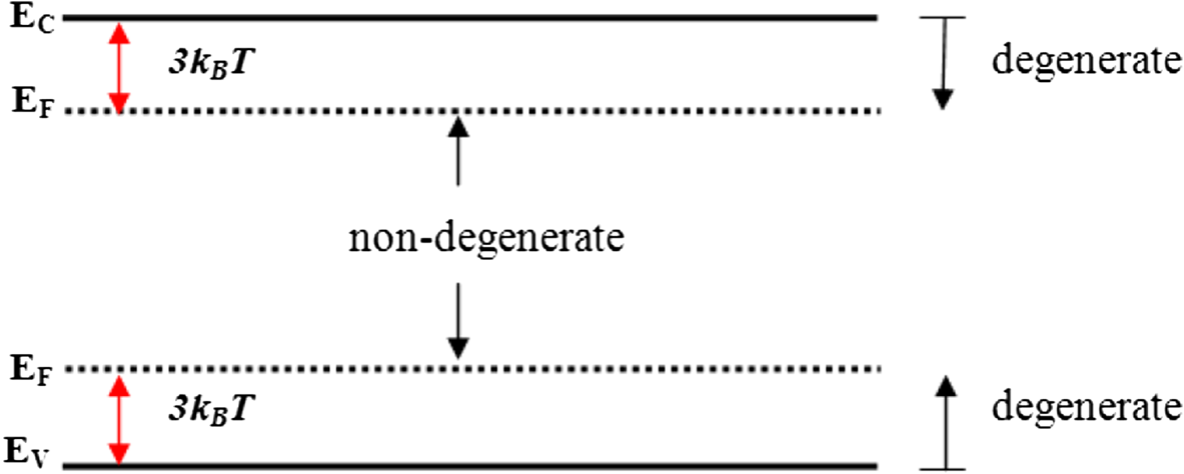
Energy band diagram showing the nondegenerate and degenerate regions.

The definition of nondegenerate and degenerate regimes of GNR capacitance maybe understood through the position of Fermi energy, *E*_*F*_ level in the energy band diagram, as illustrated in Figure 1. It is referred to as a nondegenerate regime when the Fermi level is located greater than 3 *k*_*B*_*T* in the energy gap, while the degenerate occurs if the Fermi level is located at 3 *k*_*B*_*T* within the energy band from either band edge.

The quantum capacitance at charge neutrality is calculated by differentiating the carrier density with respect to the states of energy level, resulting in the following:

(15) $$C_{Q}=\frac{e^{2}}{4\pi }\sqrt{\frac{2E_{g}}{B\left(2E-E_{g}\right)}}e^{\left(E_{c}-E_{F}/k_{B}T\right)}$$

At room temperature, the quantum capacitance in nanoscale GNRs is considered in series with the insulator capacitance. Therefore, the total gate capacitance *C*_*G*_ is given by the following [22]:

(16) $$C_{G}=C_{ins}C_{Q}/\left(C_{ins}+C_{Q}\right)$$

with *C*_*ins*_ as the gate insulator capacitance obtained as follows [23]:

(17) $$C_{ins}=N_{G}\kappa \epsilon_{0}\left(\frac{W}{t_{ins}}+\alpha \right)$$

where *N*_*G*_ is the number of gates (1 for the single-gate geometry and 2 for the double-gate geometry), *κ* is the relative dielectric constant of the gate insulator, *t*_*ins*_ is the thickness of the gate insulator, *W* is the ribbon width, and *α* ≈ 0 is a dimensionless fitting parameter.

An accurate and precise current-voltage (*I*_*D*_-*V*_*D*_) characteristic of uniaxial strained AGNRs can be obtained by including the quantum capacitance due to the quantum confinement effect. In general, the drain current, *I*_*D*_ as a function of the drain voltage, *V*_*D*_, and gate voltage, *V*_*GS*_ is given as follows:

(18) $$I_{D}=\frac{\mu_{\mathrm{eff}}C_{G}}{2L}\left[\frac{2\left(V_{GS}-V_{T}\right)V_{D}-{V_{D}}^{2}}{1+V_{D}/V_{C}}\right]$$

for 0 ≤ *V*_*D*_ ≤ *V*_*Dsat*_

where *V*_*T*_ is the threshold voltage and *V*_*Dsat*_ is the drain voltage at which the drain carrier concentration becomes maximum, consistent with the drain saturation current. *V*_*c*_ is the critical voltage that is much smaller than the drain voltage enhancing the role of velocity saturation in the nanochannel.

All the carriers in the channel travel at the saturation velocity by the onset of the current saturation, where the electric field is extremely high. The saturation current, *I*_*Dsat*_, is given by the following:

(19) $$I_{\mathrm{Dsat}}=C_{G}\left(V_{GS}-V_{T}-V_{\mathrm{Dsat}}\right)v_{sat}$$

for *V*_*D*_ ≥ *V*_*Dsat*_

Equations 18 and 19 must reconcile at the onset of current saturation. This reconciliation gives *V*_*Dsat*_ and *I*_*Dsat*_ the following expressions:

(20) $$V_{\mathrm{Dsat}}=V_{C}\left[\sqrt{1+\frac{2\left(V_{Gs}-V_{T}\right)}{V_{C}}}-1\right]$$

(21) $$I_{\mathrm{Dsat}}=\frac{1}{2}\frac{C_{G}\mu_{\mathrm{eff}}}{L}{V_{\mathrm{Dsat}}}^{2}$$

## Results and discussion

**Figure 2:**
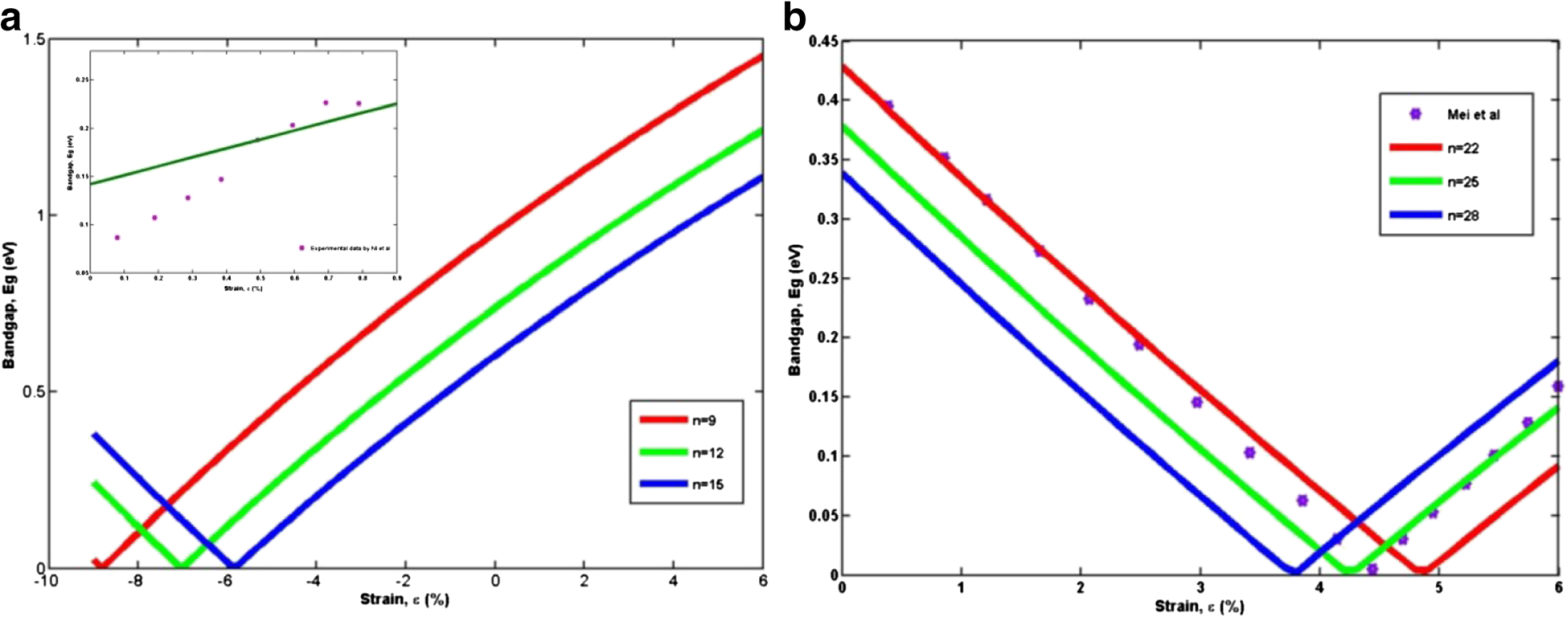
**The calculated energy bandgap *E***_***g ***_**under various uniaxial strain *ϵ *for two different families. (a) ***n* =3 *m* and **(b) ***n* =3 *m* +1.

**Figure 3:**
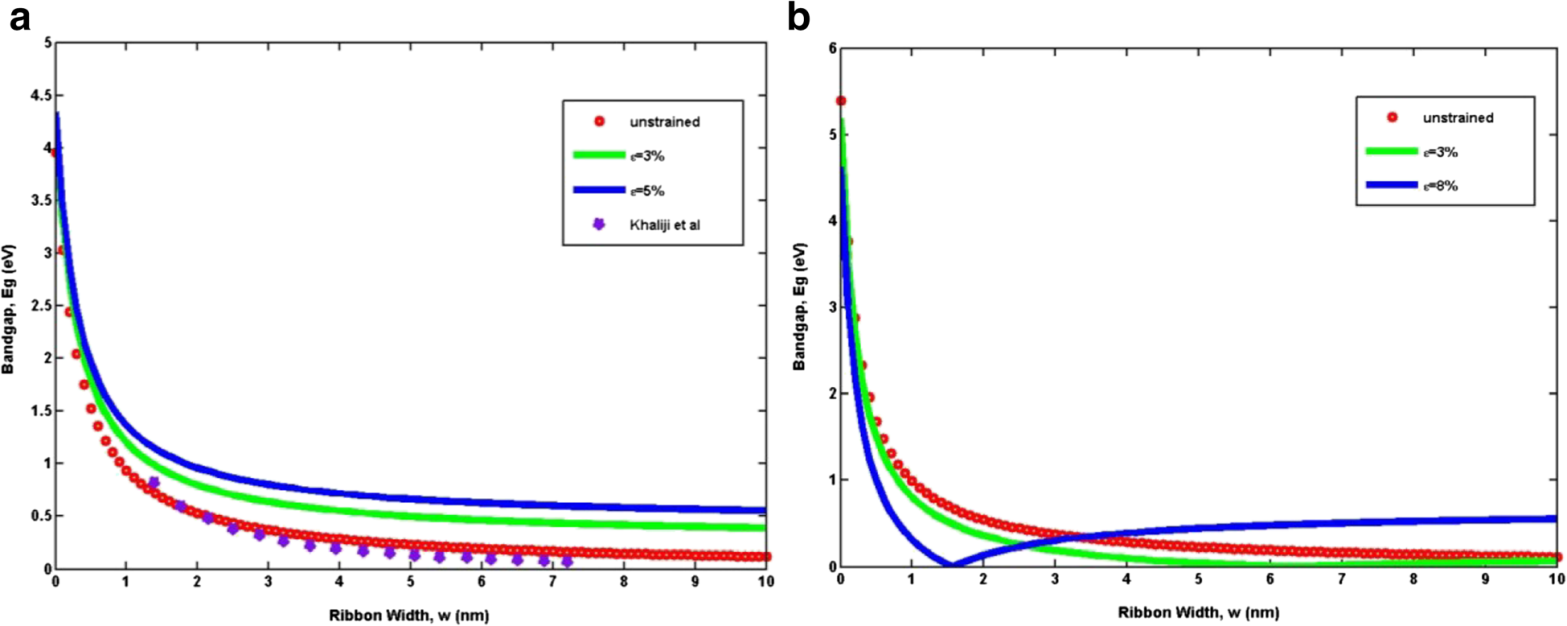
**The plot of ribbons' width versus the energy bandgap for two different families. (a) ***n* =3 *m* and **(b) ***n* =3 *m* +1. The comparison with the calculation based on density functional theory by Khaliji et al. [18] shows excellent agreement.

To clearly present the energy gap modulation due to uniaxial strain, the variations of bandgap energy for *n* =3 *m* and *n* =3 *m* +1 AGNRs as a function of strain are shown in Figure 2 with good agreement with the published data [19,24]. The calculated results for unstrained and strained AGNRs are compared. It can be observed that the effective energy bandgaps are modified in a periodic zig-zag pattern for both families and there is distinct behavior between the two families. This phenomenon can best be explained by the shift of the Fermi point perpendicular to the allowed *k* lines. When a uniaxial strain is applied, the Fermi point deviates from *K* and hence makes some bands towards or away from the Fermi point [25]. In addition, the allowed lines for both families of AGNRs have different crossing situations with the *K* point, resulting in a different energy gap [17]. For the purpose of further evaluation, the dependence of the bandgap as a function of ribbon width is depicted in Figure 3 with remarkably good agreement compared to the published data. In general, *E*_*g*_ decreases smoothly as the width of AGNRs increases, independent of the family structure. This observation can be understood by the weaker confinement in the width direction. It is worth noting that unlike the *n* =3 *m* family of AGNRs which are semiconducting, the *n* =3 *m* +1 AGNRs family could lead to a semiconductor-metal-semiconductor transition at *ϵ* =8% due to the subband spacing effect [26], as displayed by the turning point in Figure 2b.

**Figure 4:**
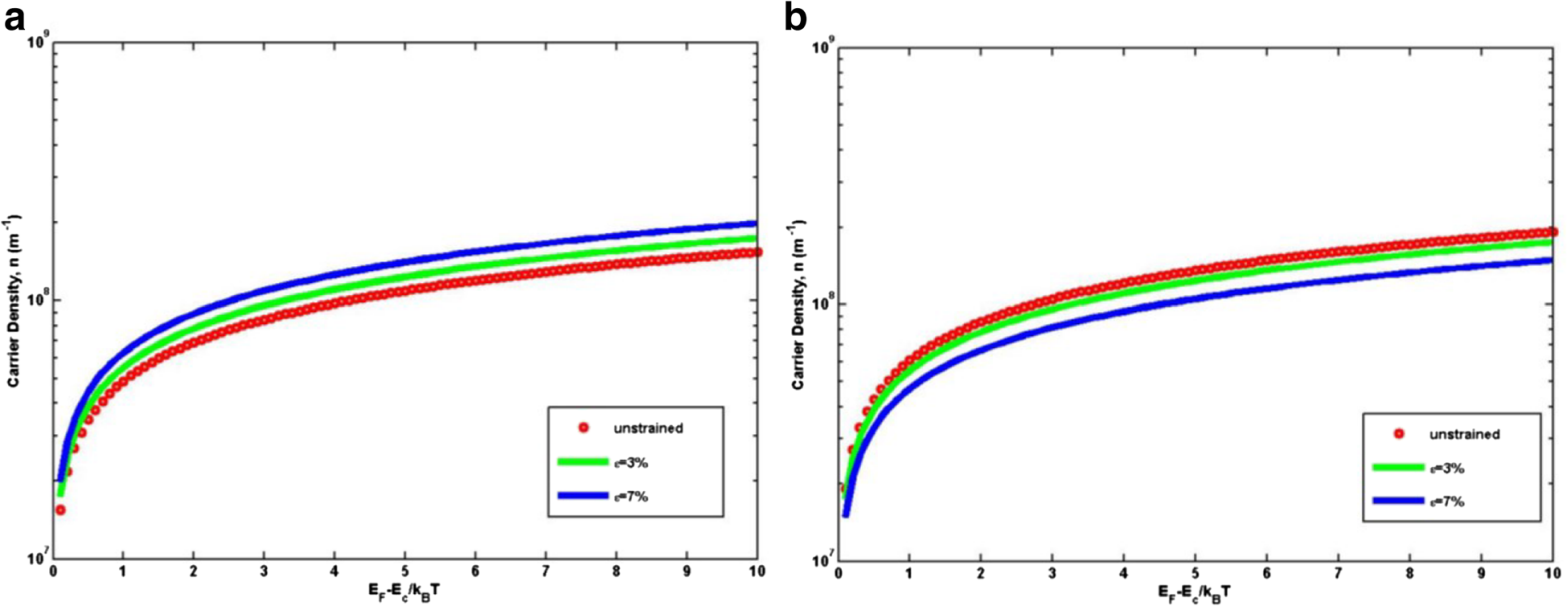
**Variation of carrier density at room temperature in AGNRs.** The variation of carrier density at room temperature in AGNRs in respect to the normalized Fermi energy for two different families **(a) ***n* =3 *m* and **(b) ***n* =3 *m* +1.

**Figure 5:**
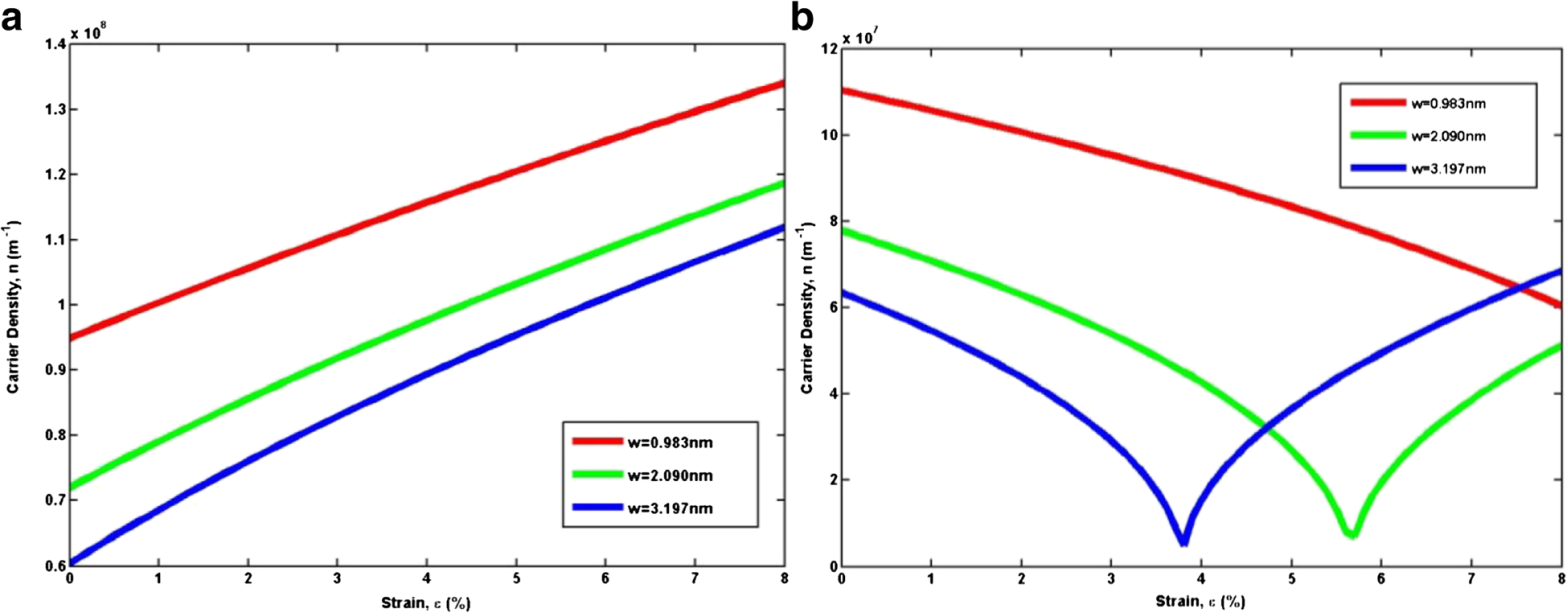
**Carrier density at room temperature in AGNRs.** Carrier density at room temperature in AGNRs for different ribbon widths as a function of uniaxial strain for two different families **(a) ***n* =3 *m* and **(b) ***n* =3 *m* +1.

Figure 4 plots the analytical carrier density at room temperature as a function of the normalized Fermi energy *η*_*F*_ at different magnitudes of strain. While the nondegenerate case has a strict linear curve (in logarithmic scale) with a high slope, the degenerate carrier density has a quasi linear curve and a reduced slope. More precisely, the slope of log(*n*) in the degenerate case is not constant but rather gradually decreases with *E*_*F*_ − *E*_*c*_/*k*_*B*_*T*. The influence of uniaxial strain on the carrier density of AGNRs is significant and quantitatively different for the two families. These figures show that for *n* =3 *m* AGNRs, uniaxial strain increases the carrier density, while on the contrary, *n* =3 *m* +1 AGNRs show a reduction in carrier density upon strain. Figure 5 plots the dependence of carrier density of different widths on the uniaxial strain at room temperature. The AGNRs with narrow ribbon width exhibit large charge modulation due to the existence of a gap, and the effect of the uniaxial strain on the characteristics of the carrier density of AGNRs shows family behavior. The carrier densities of the two families of AGNRs change accordingly to the magnitude of the strain, but for *n* =3 *m* +1 AGNRs, the carrier density does not change linearly, as in *n* =3 *m* AGNRs. Instead, one can observe turning points for *w* =2.090 nm and *w* =3.197 nm, as supported by previous observations in Figures 2 and 3.

**Figure 6:**
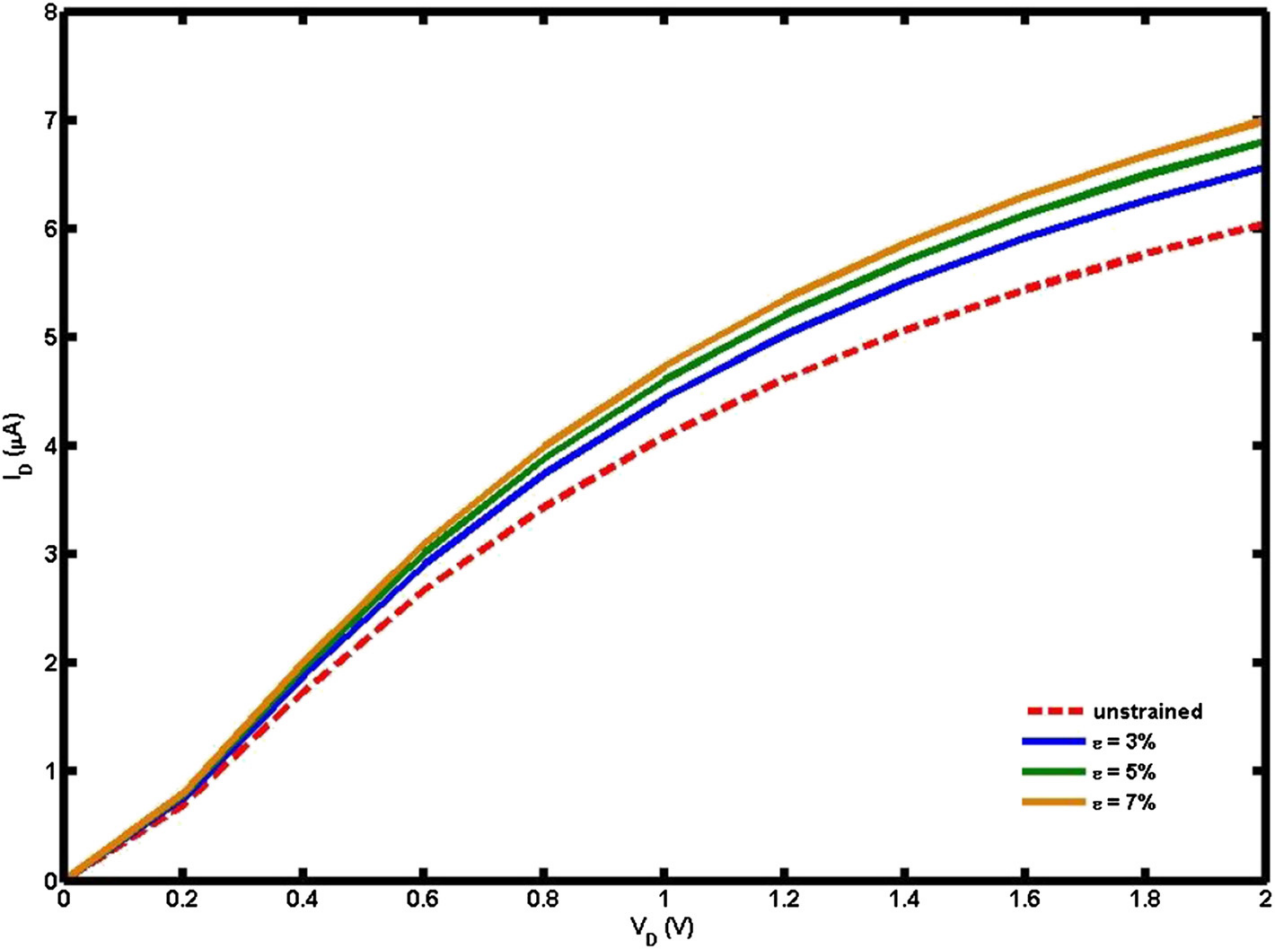
***I***_***D***_**-*V***_***D ***_**characteristic of uniaxial strained AGNRs model for different strain effects compared to unstrained AGNRs.**

Downscaling of the device dimensions has stimulated extensive efforts to further reduce the gate oxide thickness when a strong quantum confinement effect is expected. Based on the energy band structure, the analytical model of quantum capacitance of uniaxial strained *n* =3 *m* AGNRs is derived as in Equation 15 to achieve a better understanding of the atomic behaviors. Figure 6 displays the dependence of quantum capacitance on the strain effect for *n* =3 *m* AGNRs of several ribbon widths. As can be seen from the plot, the quantum capacitance increases linearly with the increase of strain. The obtained small values of the quantum capacitance at lower strain are attributed to low DOS characterization of the atomically thin quasi 1D channel [27]; the further reduction of the DOS is due to quantum confinement boundary conditions in the AGNRs' transverse direction. It is also important to note that the quantum capacitance significantly increases with the decrease in the size of ribbon width, which is a direct consequence of energy bandgap widening.

**Figure 7:**
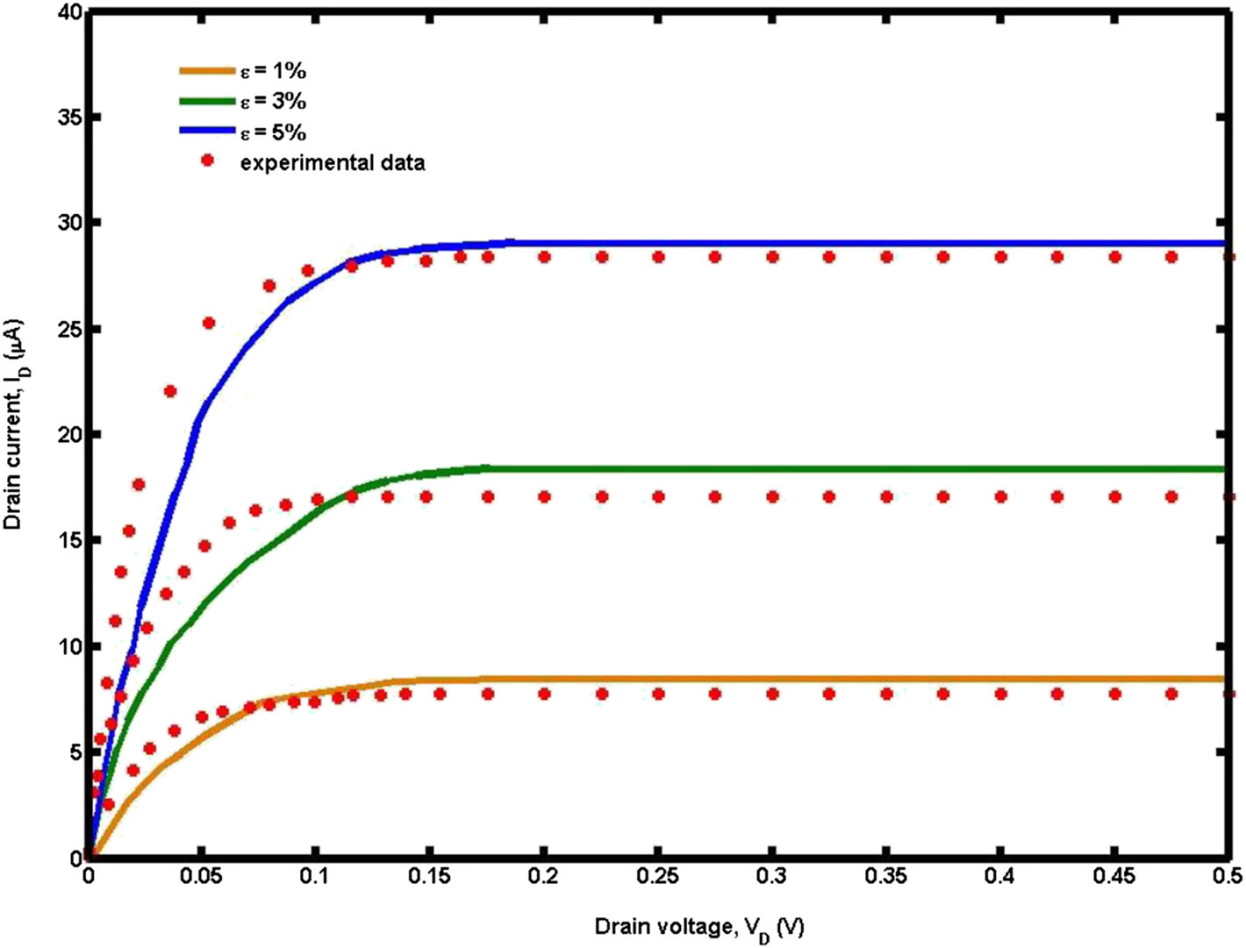
**The *I***_***D***_**-*V***_***D ***_**characteristic of uniaxial strained AGNRs compared to experimental data **[28]**.**

In order to validate the proposed analytical model, the MATLAB simulation results were compared with the experimental data [28] for a range of strain effect of uniaxial strained *n* =3 *m* AGNRs, as demonstrated in Figure 7. A good agreement was observed with no artificial parameters used in obtaining these curves. Significant drain current reduction in the proposed model was observed, resulting from the threshold voltage shift and total gate capacitance degradation due to quantum confinement. It should be noted that the influence of quantum confinement depends on the increment of gate-source voltage. Under *V*_*GS*_ =1.2 V conditions, the drain current for the proposed model drops to 1.452 μA compared to the classical value. Meanwhile, for *V*_*GS*_ =0.8 V and *V*_*GS*_ =0.4 V, the current losses are 0.798 and 0.191 μA respectively. The energy of the microscopic particles is not constant but fluctuates around some average value due to the quantum mechanical effects. The fluctuation may introduce extra energy to pump the electrons to states with higher energy.

## Conclusions

In this paper, we have calculated the electronic band structure as well as carrier density under uniaxial strain effect for both *n* =3 *m* and *n* =3 *m* +1 AGNRs families by applying a modification to the tight-binding nearest neighbor hopping integral. We observed that for *n* =3 *m* AGNRs, the bandgap increases with an increase in the magnitude of strain but tends to reduce for *n* =3 *m* +1 AGNRs family. These phenomena are caused by the moving of the Fermi point between discrete *k* lines of allowed electronics states. In addition, it is also found that the uniaxial strain gives substantial effect to the carrier density within the two families. It is also interesting to observe a semiconductor-metal-semiconductor transition phase at *ϵ* =8% for the *n* =3 *m* +1 AGNRs family. While the introduction of strain imposes changes in the bandgap and current values, the incorporation of quantum confinement effect also results in dramatic reduction in the drain current performance. The discrepancies between the classical calculation and quantum calculation can be best explained by the threshold voltage shift and total gate capacitance degradation due to quantum confinement. Our analytical findings provide critical insight into the importance of quantum confinement for nanoscale GNRs' FET, and the proposed model gives a better assessment of nanoscale GNRs' FET performance.

## Competing interests

The authors declare that they have no competing interests.

## Authors’ contributions

ESK wrote the manuscript, contributed to the design of the study, performed all the data analysis, and participated in the MATLAB simulation of the proposed device. RI participated in the conception of the project and improved the manuscript. Both authors read and approved the final manuscript.
